# Combinatorial F-G Immunogens as Nipah and Respiratory Syncytial Virus Vaccine Candidates

**DOI:** 10.3390/v13101942

**Published:** 2021-09-28

**Authors:** Ariel Isaacs, Stacey T. M. Cheung, Nazia Thakur, Noushin Jaberolansar, Andrew Young, Naphak Modhiran, Dalan Bailey, Simon P. Graham, Paul R. Young, Keith J. Chappell, Daniel Watterson

**Affiliations:** 1 School of Chemistry and Molecular Biosciences, University of Queensland, Saint Lucia 4067, Australia; a.isaacs@uq.edu.au (A.I.); s.cheung@uq.edu.au (S.T.M.C.); n.jaberolansar@uq.edu.au (N.J.); a.young4@uq.edu.au (A.Y.); n.modhiran@uq.edu.au (N.M.); p.young@uq.edu.au (P.R.Y.); k.chappell@uq.edu.au (K.J.C.); 2 The Pirbright Institute, Pirbright, Woking GU24 0NF, UK; nazia.thakur@pirbright.ac.uk (N.T.); dalan.bailey@pirbright.ac.uk (D.B.); simon.graham@pirbright.ac.uk (S.P.G.); 3 The Australian Institute for Biotechnology and Nanotechnology, University of Queensland, Saint Lucia 4067, Australia; 4 Australian Infectious Disease Research Centre, University of Queensland, Saint Lucia 4067, Australia

**Keywords:** Nipah virus, respiratory syncytial virus, vaccine, rational vaccine design, immunisation, antibody

## Abstract

Nipah virus (NiV) and respiratory syncytial virus (RSV) possess two surface glycoproteins involved in cellular attachment and membrane fusion, both of which are potential targets for vaccines. The majority of vaccine development is focused on the attachment (G) protein of NiV, which is the immunodominant target. In contrast, the fusion (F) protein of RSV is the main target in vaccine development. Despite this, neutralising epitopes have been described in NiV F and RSV G, making them alternate targets for vaccine design. Through rational design, we have developed a vaccine strategy applicable to phylogenetically divergent NiV and RSV that comprises both the F and G proteins (FxG). In a mouse immunization model, we found that NiV FxG elicited an improved immune response capable of neutralising pseudotyped NiV and a NiV mutant that is able to escape neutralisation by two known F-specific antibodies. RSV FxG elicited an immune response against both F and G and was able to neutralise RSV; however, this was inferior to the immune response of F alone. Despite this, RSV FxG elicited a response against a known protective epitope within G that is conserved across RSV A and B subgroups, which may provide additional protection in vivo. We conclude that inclusion of F and G antigens within a single design provides a streamlined subunit vaccine strategy against both emerging and established pathogens, with the potential for broader protection against NiV.

## 1. Introduction

Structure-based antigen design allows for the development of vaccine approaches that elicit targeted immune responses and has been applied for glycoproteins from viruses such as HIV, Ebola, influenza, respiratory syncytial virus (RSV), SARS-CoV-2, Nipah virus (NiV) and Hendra virus (HeV) [1,2,3,4,5,6,7,8]. Of these pathogens, henipaviruses and RSV possess two surface glycoproteins that are involved in cell attachment and membrane fusion, both of which are potential targets for vaccine and therapeutic development [9,10].

The attachment protein (G) of henipaviruses is a type II protein with a tetrameric (dimer of dimers) structure and is the primary target for vaccines and therapeutics, as it is involved in binding host cellular receptors ephrin B2/B3 [11,12,13,14]. Indeed, several protective epitopes within henipavirus G have been characterised [15,16,17,18,19] and vaccination with HeV G has yielded promising results in several animal models [20,21,22,23,24]. This lead to the licensure of an equine vaccine in Australia and the progression of this vaccine candidate into human clinical trials [25]. In contrast, the RSV G protein structure is not fully defined; however, the ectodomain is known to be heavily glycosylated, with glycans contributing to ~60% of the glycoprotein’s molecular mass [26,27,28]. Several neutralising and protective epitopes have been mapped to the central conserved domain (CCD) of RSV G [29,30,31,32], which has been implicated in binding to the fractalkine CX_3_C-chemokine receptor 1 (CX_3_CR1) [33,34,35,36]. After cellular attachment, both henipaviruses and orthopneumoviruses make use of a trimeric class I fusion protein (F) to merge viral and cellular membranes [10]. This is achieved by a structural transition of F from a metastable prefusion form to a highly stable postfusion form, driving the fusion of membranes and mediating viral entry [37,38,39]. It has been shown for both NiV and RSV that the prefusion conformation of F is most relevant for vaccine design, as this form is the main target of neutralising antibodies [37,40,41]. As such, several efforts have been made to stabilise henipavirus and RSV F proteins in the prefusion conformation through structure-based design and addition of a foldon or GCN4 trimerization domain [2,5,41].

There are currently no licensed human vaccines for henipaviruses. While G alone is an attractive target for NiV and HeV vaccine design, several studies have shown that the F glycoprotein contains neutralising epitopes and, therefore, is also a viable target [5,37,40,41,42,43]. This has been particularly evident in therapeutic development, where two cross-neutralising antibodies targeting F have been isolated and characterised [40,41]. Therefore, while henipavirus vaccines that solely target G may be effective, this could be further improved on through inclusion of F. Indeed, the pre-clinical development of a canarypox vectored (ALVAC) NiV vaccine demonstrated that vector designs including both F and G were most protective against infection in comparison to F or G alone [43]. Additionally, immunogenic evaluation of chimeric subunit vaccine designs comprising both NiV F and G glycoproteins has demonstrated more potent neutralisation of NiV than either F or G alone [5]. In contrast to henipaviruses, orthopneumovirus F is the immunodominant target and, as such, is the focus of vaccine design [44,45,46,47]. There are currently no licensed vaccines for RSV, with development hampered by the failure of a formalin-inactivated RSV vaccine in the 1960s [48]. Since then, several stabilised prefusion RSV F subunit vaccines have progressed to clinical trials [49,50]. Some RSV vaccine strategies, such as live-attenuated or vectored vaccines [51,52], subunit vaccines and virus-like particles [53], also make use of RSV G.

The characterisation of protective epitopes within NiV and RSV F and G glycoproteins has enabled the rational design of new subunit vaccines that include both antigens. Theoretically, this approach may increase the breadth and resilience of protection against viral escape mutants by providing multiple immune targets. In this study, we aimed to generate immunogens for NiV and RSV that include both the prefusion stabilised F and G glycoproteins within a single design (FxG). Using structural characterisation and in vitro analyses, our objective was to show that the FxG antigens were authentic and presented protective and neutralising epitopes within both F and G protein targets. We then sought to evaluate these antigens as subunit vaccine candidates in a mouse immunogenicity model and assess the neutralisation against potential viral escape mutants.

## 2. Materials and Methods

### 2.1. Antigen Design and Protein Expression

NiV and RSV F clamp antigens were produced as previously described [54,55,56]. Briefly, codon-optimised DNA sequences encoding the ectodomain of NiV Malaysia strain (1–483, GenBank: NP_112026.1) or RSV A2 F ectodomain (1–474, GenBank: APW29972.1) with the fusion peptide and peptide 27 deleted (Δ106-150), were synthesized by Integrated DNA Technologies (IDT). These sequences were cloned into a mammalian expression vector (pNBF; National Biologics Facility, Brisbane, QLD, Australia) under a CMV promoter and upstream of a HIV gp41 fusion core trimerization domain (molecular clamp) connected by a GSG linker, using inFusion cloning and Stellar competent cell transformation, according to the manufacturer’s protocol (TakaraBio, Shiga, Japan). The NiV FxG sequence was produced by cloning NiV G globular head domain (183–602, GenBank: NP_112027.1) into NiV F clamp expression vector, downstream of the molecular clamp sequence linked by a G_4_SG_4_SG_4_ flexible linker, using the same cloning method. RSV FxG sequence was designed in the same approach by cloning the RSV G extracellular domain (amino acids 66–297) into RSV F clamp expression vector. RSV G was made as an Fc-fusion for purification means. Here, the extracellular RSV G domain was cloned into a mammalian expression vector under CMV promoter with an immunoglobulin heavy chain V region 102 signal sequence, linked to a Fc domain by a human rhinovirus 3C (HRV3C) protease site (LEVLFQGP) for post-translational cleavage.

Plasmid DNA sequences encoding antigen sequences were transfected and expressed in the ExpiCHO-S expression system according to manufacturer’s guidelines (ThermoFisher Scientific, Waltham, MA, USA). Briefly, 1 μg plasmid DNA was transfected per 1 mL of ExpiCHO cells at a density of 1 × 10^6^ cells/mL. Seven days post-transfection, cell culture supernatant was harvested by centrifugation at 4800× *g* at 4 °C for 30 min before filter sterilisation (0.22 μm). Antigens were purified from supernatant using immunoaffinity chromatography. Here, supernatant containing secreted clamped antigens was passed through an in-house made column conjugated with a clamp-specific monoclonal antibody (mAb) HIV1281 [57], before washing the column with high salt PBS (PBS with 400 mM NaCl, pH 7.4). Proteins were eluted using a high pH buffer (100 mM Tris-HCl, 400 mM NaCl, 5 mM EDTA, 20 mM DEA, pH 11.5) and fractions were neutralised with a 1:1 *v/v* ratio of 1 M Tris pH 6.8. Protein was concentrated and buffer exchanged into PBS (Merck Amicon, Burlington, MA, USA) and protein concentration was determined using NanoDrop One at 280 nm (ThermoFisher Scientific).

The codon optimised stalk and ectodomain of NiV G (NP_112027.1; amino acids 71–602) was cloned into the pHLSec expression vector using AgeI and KpnI to fuse an N-terminal signal peptide and HIS-tag. The resulting cDNA (pNiVsG) was transfected into HEK293T cells in a roller culture using PEI transfection reagent and cultured in 3 L media containing heat inactivated FBS (F7524, Sigma-Aldrich, St. Louis, MO, USA). Supernatant was harvested 7 days post transfection and centrifuged twice at 4000 rpm for 20 min to remove cellular debris. The supernatant was filtered through a 0.45 μm filter and dialysed overnight before the sG was purified using NI IDC HiTrap HP affinity columns, eluted with 300 mM imidazole diluted in PBS to obtain protein fractions. Pooled fractions were dialysed overnight into PBS supplemented with 0.2 mM PMSF protease inhibiter and filtered through a 0.22 μm filter. The resulting sG protein concentration was quantified using a Pierce BCA Protein Assay Kit (ThermoFisher Scientific, Waltham, MA, USA) and by Coomassie staining, before being aliquoted and stored at −80 °C.

Soluble RSV G (RSV sG) was produced by cleaving the Fc-tag using HRV3C protease [58]. Briefly, RSV G-Fc was incubated with HRV3C protease at a *w*/*w* ratio of 20:1 with 50 mM Tris-HCl, 150 mM NaCl and 1 mM EDTA for 48 h at 37 °C. RSV sG was purified from Fc by size-exclusion chromatography (SEC) on a Superose 6 Increase 10/300 GL column (GE Healthcare, Chicago, IL, USA). Fractions containing RSV sG were combined and concentrated (Merck Amicon, Burlington, MA, USA). All NiV and RSV specific mAbs used in this study were produced in-house in the ExpiCHO system as previously described [59]. For reference, a summary of the antibodies used is provided in Table 1.

### 2.2. In Vitro Protein Characterisation by SDS-PAGE and SEC

Antigens were assessed for purity and molecular weight by loading 5 μg of boiled protein on a 4–12% SDS-PAGE under reducing conditions (100 mM dithiothreitol). Gels were stained in Coomassie brilliant blue R-250 for 1 h and destained in 35% methanol and 10% acetic acid. The oligomeric state of purified antigens was determined by SEC. 50–100 μg of protein was loaded on a calibrated Superose 6 Increase 10/300 GL column (GE Healthcare, Chicago, IL, USA) in a 500 μL loop. Fractions of 1 mL were collected in regard to retention volumes with peak absorbance values. Data were normalised as relative mAU based on the highest absorbance value observed per run.

### 2.3. ELISAs

Antigens were assessed for mAb binding via ELISA. Here, 2 μg/mL of antigen was coated on Nunc MaxiSorp ELISA plates (ThermoFisher, Waltham, MA, USA) and incubated overnight at 4 °C. All wells were blocked with 150 μL of blocking buffer (5% KPL milk diluent solution (SeraCare, Milford, MA, USA ) in PBS with 0.1% Tween20 (PBST)) for 1 h at room temperature. Plates were then probed with a serial dilution of mAb in blocking buffer for 1 h at 37 °C. Plates were washed thrice in water before addition of goat anti-human HRP-conjugated secondary antibody (Sigma Aldrich) diluted 1:2500 in blocking buffer for 1 h at 37 °C. Plates were washed as before prior to being developed with 50 μL/well of TMB chromogen solution (Life Technologies, Carlsbad, CA, USA) for five minutes. Substrate reactions were stopped by addition of 25 μL/well of 1 M H_2_SO_4_ before reading plate absorbance at 450 nm. Data were plotted with background binding against PBS coated wells subtracted and a one-site specific model fitted on GraphPad Prism 9 to calculate dissociation constants (K_d_). To assess binding of mAbs to RSV sG, mAbs were first biotinylated using EZ-Link Sulfo-NHS-Biotin (ThermoFisher, Waltham, MA, USA) as per manufacturer’s instructions. Binding was assessed as above except HRP-conjugated streptavidin (ThermoFisher) was used as a secondary antibody.

Antigen-specific IgG from mouse serum samples was measured by ELISA as described above. Here, titrated mouse serum samples were incubated on antigen-coated blocked plates for 1 h at 37 °C. ELISAs were performed as above, expect HRP-conjugated goat anti-mouse secondary (Sigma-Aldrich, St. Louis, MO, USA) was used at a 1:2500 dilution. Endpoint titre was calculated as the serum dilution giving an absorbance above the mean plus 3 standard deviations of control wells containing no serum sample.

### 2.4. Negative Stain Transmission Electron Microscopy (TEM)

SEC purified antigens were deposited onto carbon-coated copper grids (ProSciTech, Kirwan, QLD, Australia) at approximately 10 μg/mL and stained with 1% (*w*/*v*) uranyl acetate for 2 min. Grids were imaged using a Hitachi HT7700 at 120 KeV and images were acquired using AXT 2kx2k CMOS. Subsequent micrograph processing was conducted using Relion3.1 software and contrast transfer functions of the images were corrected using CTFFIND. Particles were selected manually (3414 particles for NiV FxG) in 25,000× magnification followed by reference-free alignment and two and three-dimensional classification.

### 2.5. Animal Immunisation

This study was conducted in accordance with the University of Queensland Animal Ethics Committee (AEC) approval (AEC SCMB/558/17, approved 27 February 2018). Female BALB/c mice aged 5–8 weeks were sourced from the Australian Resource Centre. Mice were house at the University of Queensland Biological Research Facility in individually ventilated, HEPA-filtered cages. Mice (n = 5) were immunised with 5 μg antigen or PBS formulated with 50 μg Alhydrogel (Brenntag, Essen, Germany) via intramuscular injection in the hind-leg muscle under anaesthesia. For groups immunised with a combination of prefusion F and G antigens (F + G), a 3:1 molar ratio of F trimer to G monomer was used. The vaccination regimen included three- and six-week booster immunisations, with blood samples collected via the tail vein one day prior. Terminal bleeds were collected three weeks after the final booster via cardiac puncture. Blood samples from all timepoints were allowed to coagulate overnight at 4 °C before harvesting serum by centrifugation at 10,000× *g* for 10 min at 4 °C.

### 2.6. Micro-Fusion Inhibition Test (mFIT)

NiV mFITs were carried out as previously described [67]. Briefly, HEK293T Lenti rLuc-GFP 1–7 (effector) was transfected with 500 ng of NiV-F (pGEN2.1, Myc tag) and 500 ng of NiV-G (pGEN2.1, HA tag) viral glycoproteins using Trans-IT X2 transfection reagent (Mirus Bio, Madison, WI, USA). Two days post-transfection, sera from vaccinated mice were incubated with 100 µL of 2 × 10^5^ cells/mL effector cells at a starting dilution of 1:10 and titrated 2-fold and incubated for 1 hr at 37 °C with 5% CO_2_. HEK293T Lenti rLuc 8–11 (target) cells, which endogenously express the cellular receptor for NiV (ephrin-B2), were then co-cultured at 100 µL 2 × 10^5^ cells/mL and incubated for 18 h at 37 °C with 5% CO_2_. To quantify Renilla luciferase units (RLU), media was removed from all wells and replaced with 60 µL of Coelenterazine-H, 1 µM (Promega) diluted 1:400 with PBS. Plates were incubated in the dark for 2 min then read on a GloMax Multi + Detection System (Promega, Madison, WI, USA) and CSV files were exported onto a USB flash drive for analysis. Inhibition of fusion was calculated as the percentage that yielded 50% inhibition of fusion relative to the untreated controls (IC_50_).

### 2.7. Generating Lentiviral-Based Pseudoparticles

NiV pseudovirus particles (NiV-pps) were produced as previously described [42,67]. HEK293T cells were plated in 10 cm^2^ dishes at a density of 2 × 10^6^ cells in 10 mL of DMEM (Gibco) supplemented with 10% heat-inactivated FCS, 1 mM sodium pyruvate and 10,000 U/mL of penicillin/streptomycin (D10). The following day, cells were transfected with 1 μg p8.91 (encoding HIV-1 gag-pol), 1.5 μg pCSFLW (lentiviral backbone encoding firefly luciferase reporter) and 1 μg each of NiV-F and NiV-G full-length glycoproteins in pcDNA3.1 using lipofectamine LTX reagent (ThermoFisher) as per manufacturer’s protocol. Plates were incubated overnight at 37 °C with 5% CO_2_. The following day, media on the plates was replaced with 7 mL D10 and the plates were incubated overnight. Pseudovirus particles were harvested 48, 60 and 72 h post-transfection. Harvests were pooled and centrifuged at 2000 rpm for 10 min at 4 °C to pellet cellular debris. Target BHK cells were plated at a density of 2 × 10^4^ cells per well of a white Nunc MicroWell 96-well plate in DMEM with 5% FCS (D5). The following day, NiV-pps was titrated 3-fold on target cells in D5 media and incubated at 37 °C with 5% CO_2_ for 72 h. The remaining virus was stored at −80 °C for later use. NiV-pps luciferase reporter activity was measured by discarding supernatant and adding 50 μL/well of a 1:1 mix of Bio-Glo Luciferase Assay System (Promega) and serum-free DMEM. Plates were incubated for 10 min at room temperature before reading luminescence on a Varioskan LUX (ThermoFisher).

### 2.8. Pseudovirus Neutralisation Assay

As required, target BHK cells were plated at a density of 2 × 10^4^ cells per well of a white Nunc MicroWell 96-well plate in D5 media. The following day, mAbs or heat-inactivated sera from vaccinated mice were serially diluted 8-fold in duplicate in D5 media with a final volume of 70 μL/well of a 96 well plate. An equal volume of NiV-pps was added to wells at a dilution that would yield ~2 × 10^6^ RLU and incubated for 1 h at 37 °C with 5% CO_2_. Following this, 120 μL of virus/inhibitor mixture was transferred plates containing seeded target cells and incubated for 72 h at 37 °C with 5% CO_2_. Luciferase reporter luminescence was measured as described for virus titre analysis. Serum neutralisation IC_50_s were calculate as the inverse of the dilution that yielded 50% inhibition of NiV-pps viral entry, relative to virus only controls.

### 2.9. Plaque Reduction Neutralisation Tests (PRNTs)

PRNTs were conducted in order to measure the neutralisation capacity of immunised mice sera against RSV as previously described [54]. Briefly, Nunc 96-well flat bottom tissue culture plates were seeded with 5 × 10^4^ Vero cells/well in OptiMEM (Gibco, Gaithersburg, MD, USA) supplemented with 3% heat-inactivated FCS and incubated overnight at 37 °C with 5% CO_2_. Heat-inactivated mouse serum samples were serially diluted in duplicate before 75 plaque-forming units (PFU)/well of RSV A2 virus (produced in Vero76 cells) was added. Virus-serum samples were then incubated for 1 h at 37 °C with 5% CO_2_ and adsorbed onto plated cells for 1 h at 37 °C with 5% CO_2_. Then, 100 μL of overlay medium (M199 media (Gibco) supplemented 2% heat-inactivated FCS, penicillin-streptomycin and 1.5% medium viscosity carboxymethyl cellulose (CMC)) was added per well and plates were incubated for 3 days at 37 °C with 5% CO_2_. Plates were fixed in 80% acetone/20% PBS for 20 min at −20 °C. Plates were allowed to dry before adding 150 μL/well of blocking buffer and incubating at room temperature for 1 h. All wells were then stained with 1 μg/mL of anti-RSV F human motavizumab for 1 h at 37 °C. Plates were washed thrice in PBST before adding anti-human IR800 (Millennium Science, Mulgrave, Australia) diluted 1:2500 in blocking buffer and incubating for another hour at 37 °C. The washing step was repeated and plaques were imaged by scanning plates on an Odyssey CLX imaging system (LI-COR). Counted plaques were plotted on GraphPad Prism 9, where a 3-parameter log(inhibitor) vs. response model was fit to calculate PRNT_50_s.

## 3. Results

### 3.1. Rational Design of FxG Results in Expression of Immunogens Containing Antigenically-Sound F and G Glycoproteins

**Figure 1:**
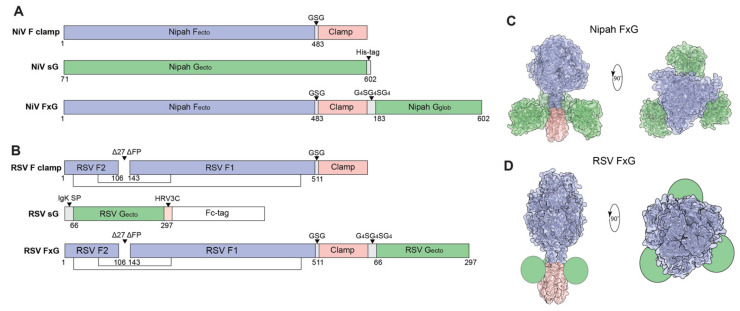
Rational design of NiV and RSV antigens. (**A**) Schematics of prefusion NiV F, NiV sG and NiV FxG antigens. F ectodomains (F_ecto_) are coloured in blue, clamp trimerization domain coloured in salmon and G ectodomain (G_ecto_) coloured in green. (**B**) Schematics of prefusion RSV F, RSV sG and RSV FxG antigens with the same colouring scheme as in (**A**). The RSV F clamp antigen contains a deletion of the fusion peptide (FP) and peptide 27. The RSV sG antigen is engineered with a human rhinovirus 3C (HRV3C) protease site (orange) to cleave C-terminus Fc purification tag (white). Proposed in silico designs of NiV (**C**) and RSV (**D**) FxG structures are modelled using F structures (NiV PDB 5EVM; RSV PDB 4MMV), G structures (NiV PDB 3D11; RSV G represented as green circles) and clamp structure (PDB 1I5Y) with the same colouring scheme as in (**A**). Structures made using UCSF ChimeraX.

**Figure 2:**
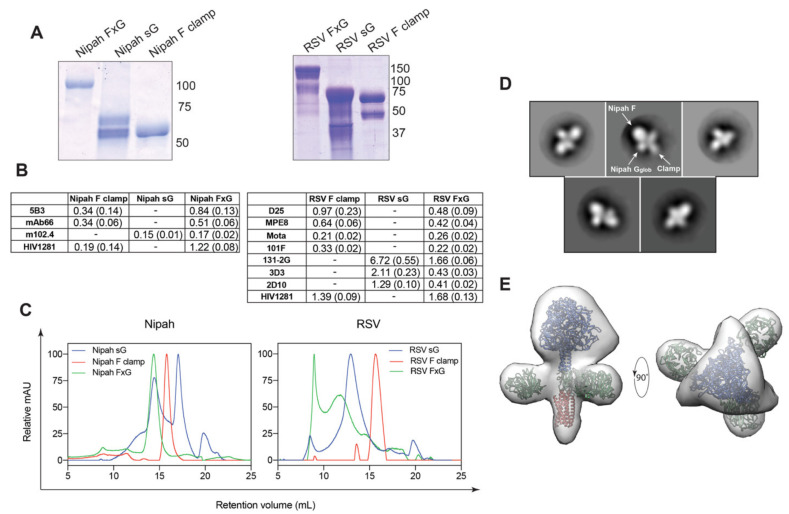
NiV and RSV FxG antigen characterisation. (**A**) SDS-PAGE of NiV (left) and RSV (right) purified antigens run under reducing conditions. Uncropped gels are provided in Figure S1. (**B**) K_d_ in nM of NiV mAbs (left) and RSV mAbs (right) against F clamp, sG and FxG antigens with standard error shown in parentheses and dashed line showing no applicability. K_d_ are calculated from indirect ELISAs of mAbs against purified proteins, shown in Figure S1. An additional clamp-specific mAb (HIV1281) is included as a control. (**C**) SEC of NiV (left) and RSV (right) antigens ran on Superose 6 Increase 10/300GL column and normalised to the highest mAU value of each run. (**D**) Representative two-dimensional class averages of NiV FxG obtained from negative stain electron microscopy with annotations depicting potential domains of FxG. (**E**) An initial ~30 Å three-dimensional model of NiV FxG antigen obtained from negative stain electron microscopy analyses with known structures of NiV F (PDB 5EVM), NiV G head domain (PDB 3D11) and clamp domain (PDB 1AIK) fitted and coloured in blue, green and red respectively.

The ectodomains of NiV F and RSV F proteins were expressed and purified as previously described [54,55,56,68]. A molecular clamp trimerization domain was incorporated at the C-terminus of NiV F (NiV F clamp) and RSV F (RSV F clamp) glycoprotein ectodomains to stabilise them in the prefusion conformation (Figure 1A,B). The extracellular domain of RSV G was linked to a C-terminal Fc-tag for purification means, which was cleaved after affinity purification by making use of a HRV3C protease site (Figure 1B), yielding soluble RSV G (sG). The full ectodomain of NiV G was expressed and purified by His-tag purification. The design of the NiV FxG antigen consisted of the F clamp sequence connected at the C-terminus of the clamp domain to the N-terminus of NiV G globular head domain via a G_4_SG_4_S linker, forming a covalently linked NiV F clamp and G chain (Figure 1A). The RSV FxG antigen was designed with the same approach, except the full extracellular domain of RSV G was included (Figure 1B). Our designs envisaged a single F trimer displaying three G domains around a central axis (Figure 1C,D). Antigens were assessed for purity and molecular size by SDS-PAGE (Figure 2A and Figure S1). Here, we observed highly pure antigens, with a molecular weight of NiV F clamp of ~65 kDa, NiV sG ~65–70 kDa and NiV FxG ~110 kDa under reducing conditions, which is consistent with the predicted monomeric molecular weights of 60, 67 and 107 kDa, respectively. Under reducing conditions, RSV F clamp migrated to a molecular weight of ~70 kDa, with an additional band corresponding to the F_1_ fragment at ~50 kDa. This is consistent with the predicted monomeric molecular weight of RSV F clamp of ~65 kDa. Based on its sequence, RSV sG has a predicted molecular weight of 25 kDa; however, it is extensively glycosylated. On an SDS-PAGE, RSV sG was ~75 kDa with a smear banding pattern observed, likely due to extensive glycosylation. This was also observed to a lesser extent for RSV FxG with an observed molecular weight of ~150 kDa, which was in line with the predicted value of ~140–150 kDa.

To probe the antigenic properties of NiV FxG proteins, ELISAs were conducted with both prefusion F- and G-specific neutralising mAbs. Here, NiV FxG was able to bind prefusion F-specific mAbs 5B3 and mAb66 with a similar affinity to NiV F clamp (Figure 2B and Figure S1). Of note, the B_max_ level of mAb66 against NiV F clamp was reduced compared to other mAbs, suggesting reduced epitope availability or presentation in the ELISA format (Figure S1B). NiV FxG was also reactive to m102.4, with a comparable affinity to NiV sG. A similar analysis was conducted for RSV, where it was observed that RSV FxG was able to bind prefusion F-specific mAbs D25 and MPE8 and pre- and post-fusion specific mAbs motavizumab and 101F with similar affinities to RSV F clamp antigen (Figure 2B). RSV FxG also bound G-specific neutralising mAbs 131-2G, 3D3 and 2D10, with improved affinities in comparison to RSV sG alone. Both RSV FxG and NiV FxG bound a clamp-specific mAb HIV1281. Next, the oligomeric state of FxG antigens was determined by SEC (Figure 2C). Here, both NiV and RSV F clamp antigens yielded a single peak, corresponding to trimeric glycosylated molecular weight of ~200–220 kDa. NiV sG yielded two major peaks, likely corresponding to monomeric and higher oligomeric states. RSV sG was observed to elute as a single broad peak, likely attributed to post-translational glycosylation patterns yielding a heterogenous sample. Nipah FxG eluted as a single peak, while RSV FxG possessed two peaks, one of which is an aggregated product.

To confirm our initial in silico design of NiV FxG, we conducted single particle analysis on size-excluded proteins imaged by TEM. Here, we observed a homogenous preparation of antigens, with 2D class averages revealing domains corresponding to NiV F ectodomain surrounded by three G globular head domains (Figure 2D). From this, we were able to construct a 3D model which revealed a structure highly reminiscent of initial designs, with three G globular heads surrounding F around a three-fold central axis that is facilitated by the clamp trimerization domain (Figure 2E). Together, our in vitro analyses show that the FxG antigens are antigenically sound; the antigens are highly pure, display multiple neutralising epitopes against both F and G and form higher order oligomers. Moreover, TEM analysis revealed that NiV FxG immunogens closely resemble in silico designs.

### 3.2. FxG Antigens Elicit an Immune Response Directed against Both Protein Targets

**Figure 3:**
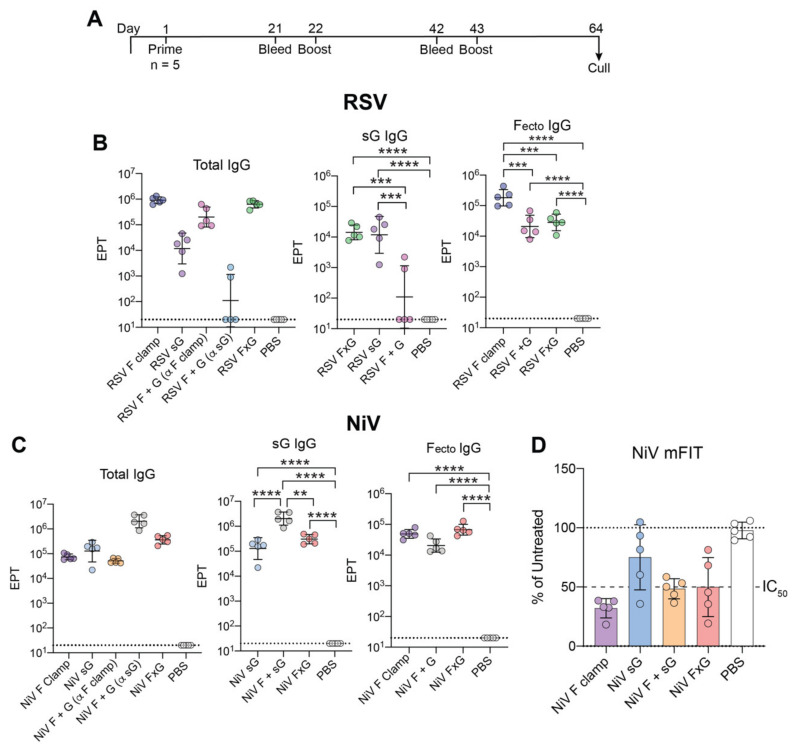
Immunogenicity of terminal bleed sera from NiV and RSV F, G, F + G and FxG vaccination in BALB/c mice. (**A**) Timeline of immunisation regimen employed. (**B**) Endpoint titres (EPT) of each RSV antigen group against vaccinated antigen/s (total IgG), RSV sG & RSV F ectodomain (F_ectdo_, DsCav foldon). (**C**) EPTs of each NiV antigen group against vaccination antigen/s (total IgG), NiV sG & NiV F ectodomain (F_ecto_, NiV F foldon). Dotted line shows limit of detection and data is expressed as geometric mean with geometric standard deviation (SD). *p*-values calculated using a one-way Tukey’s multiple comparison ANOVA on log transformed values, where * = *p* < 0.05, ** = *p* < 0.005, *** = *p* < 0.0005 and **** = *p* < 0.0001. (**D**) NiV-mFIT of a 1:40 dilution of serum samples from respective antigen groups. Data is expressed as a percentage of the average luciferase readings seen in untreated (no serum) controls with 50% inhibition (IC_50_) indicated as a dashed line. Each sample was assayed in triplicate with group mean and SD shown.

To determine how FxG antigens may affect immunogenicity in comparison to F and/or sG antigens alone, groups of 5 BALB/c mice were immunised with a total of 5 μg antigen with 50 μg Alhydrogel per dose (Figure 3A). After three doses, sera were analysed for binding against vaccine antigen, the F ectodomain, sG or the clamp domain (Figure 3). To exclusively measure NiV and RSV F ectodomain specific responses, we made use of foldon-stabilised F antigens (NiV F foldon or RSV DsCav) in order to circumvent any clamp-specific responses. Most animals immunised with both F and G proteins (as FxG or co-administered) elicited antibody responses against both targets, with the exception of three animals in the RSV F + G that did not elicit a response against RSV sG (Figure 3B). Of interest, RSV FxG elicited a similar level of a G-specific response compared to RSV sG group. RSV F clamp elicited the highest F-ectodomain specific response, with significant differences observed in comparison to both RSV F + G and RSV FxG (Figure 3B). In contrast to RSV, NiV F + G elicited the highest response against NiV sG in comparison to all other groups (Figure 3C). Similar levels of NiV F-ectodomain specific and clamp-specific responses were observed for all NiV groups (Figure 3C and Figure S2).

Previous studies have demonstrated that inhibition of syncytia formation and cell fusion may correlate significantly with functional immunity [42,67]. Using an established micro-fusion inhibition test (mFIT), we determined whether sera from vaccinated groups were able to inhibit fusion between effector cells transfected with NiV viral glycoprotein/s (NiV F and G) and target cells expressing viral entry receptors (Figure 3D and Figure S2). Here, we observed that all NiV vaccination groups were able to effectively inhibit fusion, with a more potent response observed for groups vaccinated with NiV F (Figure 3D).

### 3.3. Two Vaccine Targets Allow for More Efficient Neutralisation of a NiV Pseudovirus Mutant

**Figure 4:**
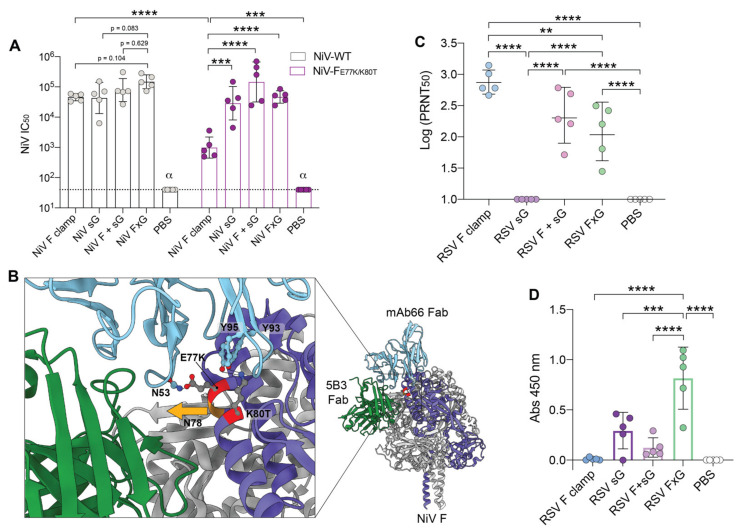
Terminal bleed sera neutralisation of NiV pseudovirus and RSV. (**A**) NiV antigen vaccinated sera neutralisation of WT-NiV or NiV-F_E77K/K80T_ epitope-knockout pseudovirus. Dotted line shows limit of detection. (**B**) Structural representation of NiV-F_E77K/K80T_ mutations. NiV F monomer coloured in purple with E77K and K80T mutations highlighted in red. 5B3 Fab (PDB 6TYS) coloured in green and mAb66 Fab (PDB 6T3F) coloured in cyan. Residues of 5B3 and mAb66 that interact with E77 or K80 on NiV-F are annotated & displayed as stick figures. A potential glycan site is formed on NiV F N78 (orange) with the proposed glycan orientation depicted by the orange arrow. (**C**) Serum samples from RSV groups tested in a plaque reduction neutralisation test (PRNT) against RSV A2 virus. In (**A**,**C**), data represents geometric mean with geometric SD and *p*-values calculated using Tukey’s multiple comparison ANOVA on log transformed values, where ** = *p* < 0.005, *** = *p* < 0.0005 and **** = *p* < 0.0001. α denotes *p* < 0.0001 against all groups unless otherwise specified. (**D**) RSV serum samples (1:10 dilution) tested for reactivity against the CCD of RSV G (MBP-CCD) in an indirect ELISA. Data shows mean with SD, with background binding against MBP subtracted. *p*-values calculated using a one-way Tukey’s multiple comparison ANOVA, where *** = *p* < 0.005 and **** = *p* < 0.0001. Non-significant comparisons are not denoted.

The efficacy of NiV FxG vaccine was evaluated in a NiV pseudovirus (NiV-pps) neutralisation assay. Here, we observed that all immunised groups were able to neutralise NiV-pps, with the FxG group eliciting the highest neutralising immune response, with lower *p*-values compared to NiV F clamp (*p* = 0.104) and NiV sG (*p* = 0.08) (Figure 4A). Interestingly, while NiV F + G elicited the highest G-specific response, this did not translate to improved neutralisation of NiV-pps.

To further probe the functional immunity generated against NiV, we used the published structures of NiV in complex with mAbs 5B3 and mAb66 to design two mutant NiV F constructs with key protective sites altered (Figure 4B) [40,41]. The first mutant, NiV-F_P52N/K55E_, included the previously identified charge swap K-E escape mutation at position 55, which has been shown to confer resistance to neutralisation by the prefusion specific 5B3 mAb against live NiV [41]. To further reduce the binding of 5B3-like mAbs, we also included an additional mutation at position 52 (P52N), which introduced a potential N-linked glycan site due to the presence of a downstream native serine residue at position 54. The second mutant, NiV-F_E77K/K80T_, contained changes at positions 77 and 80, which also introduced a charge reversal and potential N-linked glycan site, but in this case within the binding site of mAb66. Of interest, we observed that these mutants generated productive pseudovirus, with higher titres observed for the NiV-F_E77K/K80T_ mutant in comparison to wild-type NiV-pps (Figure S3). While the NiV-F_P52N/K55E_ pseudovirus knockout resulted in complete abrogation of 5B3 neutralisation, no significant changes in neutralisation were observed against NiV F antisera or mAb66 in comparison to wild-type NiV-pps (Figure S4). Interestingly, although NiV-F_E77K/K80T_ had been designed to evade recognition by mAb66, NiV-pps produced with this construct showed significantly reduced neutralisation and sensitivity to both mAb66 and 5B3 (Figure S4). Furthermore, neutralisation conferred by NiV F clamp vaccination was reduced by ~35-fold against NiV-F_E77K K80T_ in comparison to wild-type NiV-pps (*p* < 0.0001) (Figure 4A). However, we observed that groups vaccinated with NiV G, F + G or FxG retained a similar neutralisation capacity against NiV-F_E77K/K80T_ as was observed against wild-type NiV-pps (Figure 4A). These results suggest that the E77K K80T mutations alter an immunodominant epitope within NiV F, as these mutations are able to significantly dampen neutralisation by both 5B3 and mAb66 and result in a significant decrease of neutralisation by F anti-sera. Overall, these findings demonstrate that the NiV FxG strategy provides a neutralising immune response against both F and G glycoproteins, thereby eliciting a more resilient neutralising response.

### 3.4. RSV FxG Elicits Immune Responses against the Cross-Protective Central Cysteine Domain

The effectiveness of RSV FxG in neutralising RSV A2 was evaluated in a PRNT. Here, we observed that RSV F clamp elicited the highest PRNT_50_ against RSV, performing significantly better than RSV FxG (Figure 4C), likely due to immunisation with a higher relative amount of F antigen as a result of matched total antigen dose between groups. To expand, a 5 μg dose of RSV F clamp is equivalent to ~25 pmol of F trimer, whereas a 5 μg dose of RSV FxG equals to ~18 pmol of F trimer, resulting in a ~28% decrease in F content. Since neutralisation of RSV through G-specific responses is not effectively measured in a PRNT format, we sought to evaluate the RSV G CCD-specific responses, which has been previously shown to elicit neutralising and protective immune responses [29,30,31,32,33,34,35,36]. To test this, we produced the RSV G CCD linked to maltose-binding protein (MBP), which was highly pure and reactive to CCD-specific mAbs 3D3 and 2D10 (Figure S5). When quantifying CCD-specific immune responses, we observed that the RSV FxG group elicits the strongest immune response against the CCD, performing significantly better than all other groups (Figure 4D).

## 4. Discussion

The high mutation rate of RNA viruses poses significant challenges for vaccine design and therapeutic development, particularly for approaches that make use of a single target. Here, we describe a subunit vaccine strategy for NiV that includes both F and G glycoproteins. This design results in antigenically sound immunogens that are reactive to antibodies that target neutralising epitopes within both glycoproteins. Our findings demonstrate that the FxG strategy provides a resilient immunity against a potential NiV F escape mutant that can partially escape neutralisation of both 5B3 and mAb66. In line with previous studies that made use of both F and G in vaccine designs [5,43], our results indicate that a vaccine candidate against NiV that targets both glycoproteins, such as FxG, provides a broader antibody response in comparison to F or G alone by increasing the number of neutralising epitopes present, leading to immune responses that are more resilient to viral escape.

In this study, we also produced two novel epitope knockout F constructs that produced productive pseudoviruses. We found that the 5B3 resistant NiV-F_P52N/K55E_ mutant was still able to be effectively neutralised by F antisera, suggesting that this epitope is not an immunodominant epitope in mice and that NiV F clamp vaccination elicits antibodies against other neutralising epitopes within F. Unexpectedly, the mAb66 KO design, NiV-F_E77K/K80T_, resulted in significant reduction of neutralisation by both 5B3 and mAb66. Here, the E77K mutation switches the charge of a surface exposed residue within F that is positioned close to the complementary determining region (CDR)-H2 of 5B3 [41]. Additionally, it is likely that the K80T mutation, which introduces a potential N-linked glycosylation site (^78^NYT^80^), may alter 5B3 recognition due to the potential glycan addition at N78, which sits at the trimeric interface (Figure 4C). Structural analyses revealed that the 5B3 quaternary epitope spans two NiV F protomers, facilitated by residues S52 and Y57 in the CDR-H2. Addition of a glycan at position N78 within the upstream helix of a neighbouring F protomer may perturb this quaternary interface and therefore affect 5B3 binding. When evaluating mAb66 binding, both E77K and K80T directly interfere with the mAb66 interface, particularly Y93 and Y95 within CDR-L3, which have been previously deemed as key residues for binding [40]. Most importantly, the F_E77K/K80T_ mutations reduced the effectiveness of F anti-sera 35-fold in comparison to wild-type, a result that highlights that multiple targets in NiV vaccine development are likely required to elicit immune responses that are resistant to viral escape.

In contrast to NiV where G is the immunodominant target, RSV vaccine development is currently focused on F [44,45,46,47,69]. Indeed, several studies have demonstrated the importance of F during infection, specifically that RSV entry and replication can occur in the absence of G [70,71]. Despite this, there is a body of research that has highlighted neutralising and protective epitopes within RSV G, specifically the CCD, which has been shown to interact with CX3CR1 in several studies [33,34,35,36]. Moreover, several neutralising and protective antibodies have been isolated that target the CCD within RSV G [29,30,31,32]. Immunisation of mice with RSV G immunogens has also been shown to elicit antibodies capable of blocking the interaction of G with CX3CR1 [72], providing a rationale for inclusion of the CCD in vaccine designs. In this study, we extended the FxG strategy from NiV (*Paramyxoviridae*) to phylogenetically distinct RSV (*Pneumoviridae*). Here, we demonstrated that the RSV FxG antigen is able to bind prefusion F-specific antibodies as well as RSV G CCD-specific antibodies. We observed that immunisation of mice with RSV FxG resulted in inferior neutralisation in comparison to RSV F clamp and that this is likely due to immunisation with a lower F content. Indeed, several studies have demonstrated a proportional relationship between prefusion RSV F dose and virus neutralisation titres [69,73,74]. Alternatively, it is possible that the aggregation of RSV FxG observed in size exclusion profile may obscure antibody epitopes and therefore result in reduced neutralisation. In addition, neutralisation titres were measured by traditional PRNT on immortalised cells, where neutralisation by G-specific responses is not effectively measured. This has been highlighted in several studies that show that CCD-specific antibodies do not neutralise in traditional PRNTs, yet are neutralising when assayed in human primary airway epithelial cells cultured in liquid–air interfaces and have also been shown to be protective in vivo [29,30,32,34,35,75]. As a proxy for this, we measured CCD-specific responses and observed that RSV FxG elicited the strongest response in comparison to all groups, which is likely due to the trimeric presentation of RSV G within the FxG design. Given that the CCD of G is highly conserved across RSV subgroups and drawing on from other studies [66,76,77,78], it would be of future interest to test whether the elevated responses against the CCD conferred by RSV FxG may translate to broader in vivo protection for both A2 and B1 RSV subgroups.

## 5. Conclusions

In summary, we have produced and characterised a subunit vaccine strategy that comprises both F and G glycoproteins of NiV that is broadly applicable to a phylogenetically distinct pneumovirus RSV. The FxG antigens allow for a streamlined production system of both F and G glycoproteins within a single design, providing a more economical and straightforward pathway for immunisation of two immunogens. In particular for NiV, our results highlight the importance of including multiple antigenic targets in vaccine design in order to elicit an immune response that is broadly protective and resistant to viral escape mutants.

## Figures and Tables

**Table 1:** Summary of mAbs used in this study for antigen characterisation. All mAbs are made in-house.

Target	mAb	Specificity	Reference
NiV	5B3	F	[41]
	mAb66	F	[40]
	m102.4	G	[19]
RSV	MPE8	F (Site III)	[60]
	D25	F (Site Ø)	[61]
	101F	F (Site IV)	[62]
	Motavizumab	F (Site II)	[63,64]
	131-2G	G (CCD)	[65]
	3D3	G (CCD)	[66]
	2D10	G (CCD)	[31]
Clamp	HIV1281	HIV gp41 postfusion core	[57]

## Data Availability

The data presented in this study are available within the article and supplementary material.

## Supplementary Materials

The following are available online at www.mdpi.com/article/10.3390/v13101942/s1, Figure S1:(A) SDS-PAGE of RSV and NiV antigens run under reducing conditions. M is Bio-Rad Kaleidoscope molecular marker. (B) Indirect ELISAs of mAbs against NiV and RSV antigens, Figure S2: (A) Endpoint titer (EPT) of each RSV (left) and NiV (right) groups against the molecular clamp domain, assayed against a non-specific clamp-stabilised protein (B) mFITs of NiV groups showing serial dilutions of sera from vaccinated groups as well as positive mAb controls, Figure S3: Titration of NiV pseudovirus (NiV-pps) wild-type or mutant F variants on BHK-21 cells, Figure S4: NiV pseudovirus neutralisation assays of mAbs (A) and sera (B) against wild-type NiV (WT) or NiV-FP52N/K55E mutant knockout. (C) NiV pseudovirus neutralisation assay of mAbs against NiV- FE77k/K80T mutant knockout, Figure S5: (A) SDS-PAGE of MBP and MBP-CCD purified protein run under reducing conditions. (B) Indirect ELISA of CCD-specific mAbs or non-specific control (C05) against MBP-CCD protein.
