# Supplementary material for: Combinatorial F-G Immunogens as Nipah and Respiratory Syncytial Virus Vaccine Candidates
Source: Viruses. 2021 Sep 28;13(10):1942. doi: 10.3390/v13101942 (PMC8537613; doi:10.3390/v13101942)
Supplement: Supplementary file 1 [file viruses-13-01942-s001.zip › viruses-1340996-supplementary.pdf]

viruses

Supplementary Materials

Combinatorial F-G Immunogens as Nipah and Respiratory Syncytial Virus Vaccine Candidates

Ariel Isaacs [1], Stacey T. M. Cheung [1], Nazia Thakur [2], Noushin Jaberolansar [1,3], Andrew Young [1,3], Naphak Modhiran [1], Dalan Bailey [2], Simon P. Graham [2], Paul R. Young [1,3,4], Keith J. Chappell [1,3,4] and Daniel Watterson [1,4,*]

[1] School of Chemistry and Molecular Biosciences, University of Queensland, Saint Lucia 4067, Australia; a.isaacs@uq.edu.au (A.I.); s.cheung@uq.edu.au (S.T.M.C.); n.jaberolansar@uq.edu.au (N.J.); a.young4@uq.edu.au (A.Y.); n.modhiran@uq.edu.au (N.M.); p.young@uq.edu.au (P.R.Y.); k.chappell@uq.edu.au (K.J.C.)
[2] The Pirbright Institute, Pirbright, Woking GU24 0NF, UK; nazia.thakur@pirbright.ac.uk (N.T.); dalan.bailey@pirbright.ac.uk (D.B.); simon.graham@pirbright.ac.uk (S.P.G.)
[3] The Australian Institute for Biotechnology and Nanotechnology, University of Queensland, Saint Lucia 4067, Australia
[4] Australian Infectious Disease Research Centre, University of Queensland, Saint Lucia 4067, Australia
* Correspondence: d.watterson@uq.edu.au

Citation: Isaacs, A.; Cheung, S.T.M.; Thakur, N.; Jaberolansar, N.; Young, A.; Modhiran, N.; Bailey, D.; Graham, S.P.; Young, P.R.; Chappell, K.J.; et al. Combinatorial F-G Immunogens as Nipah and Respiratory Syncytial Virus Vaccine Candidates. *Viruses* **2021**, *13*, 1942. https://doi.org/10.3390/10.3390/v13101942

Academic Editor: Christopher C. Broder

Received: 30 July 2021
Accepted: 21 September 2021
Published: 28 September 2021

Publisher's Note: MDPI stays neutral with regard to jurisdictional claims in published maps and institutional affiliations.

Copyright: © 2021 by the authors. Licensee MDPI, Basel, Switzerland. This article is an open access article distributed under the terms and conditions of the Creative Commons Attribution (CC BY) license (http://creativecommons.org/licenses/by/4.0/).

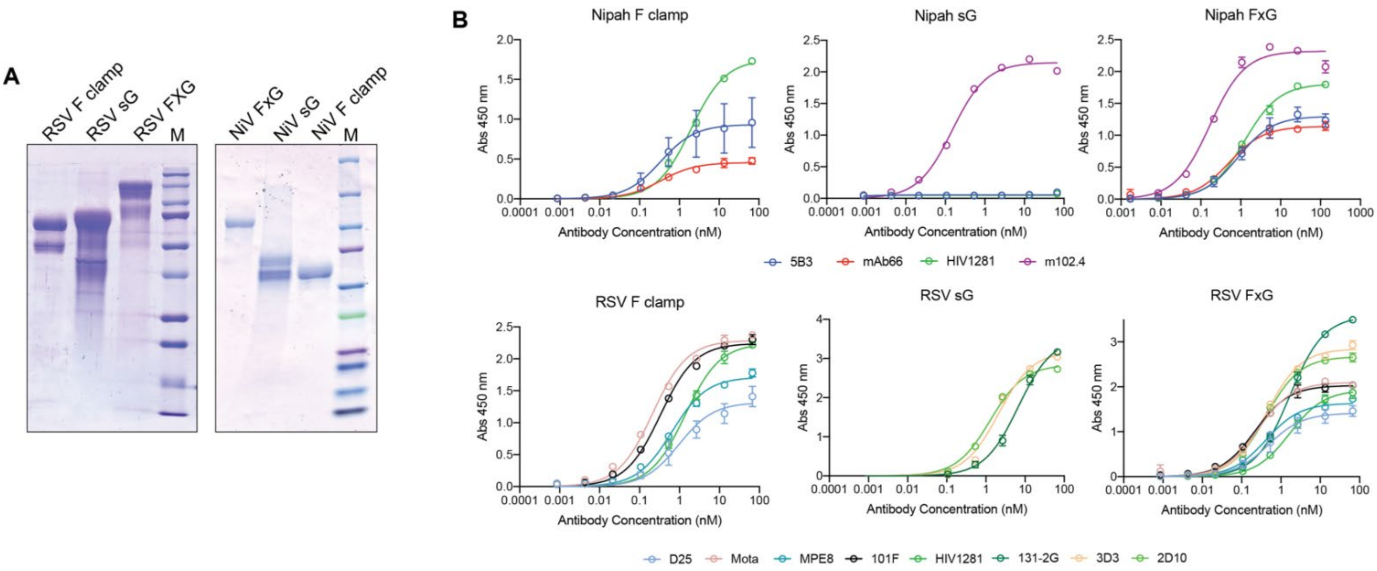

Figure S1. (**A**) SDS-PAGE of RSV and NiV antigens run under reducing conditions. M is Bio-Rad Kaleidoscope molecular marker. (**B**) Indirect ELISAs of mAbs against NiV and RSV antigens. Data shown of mean of duplicate values with standard deviation. Data fitted with one-site specific binding model to calculate K_d.

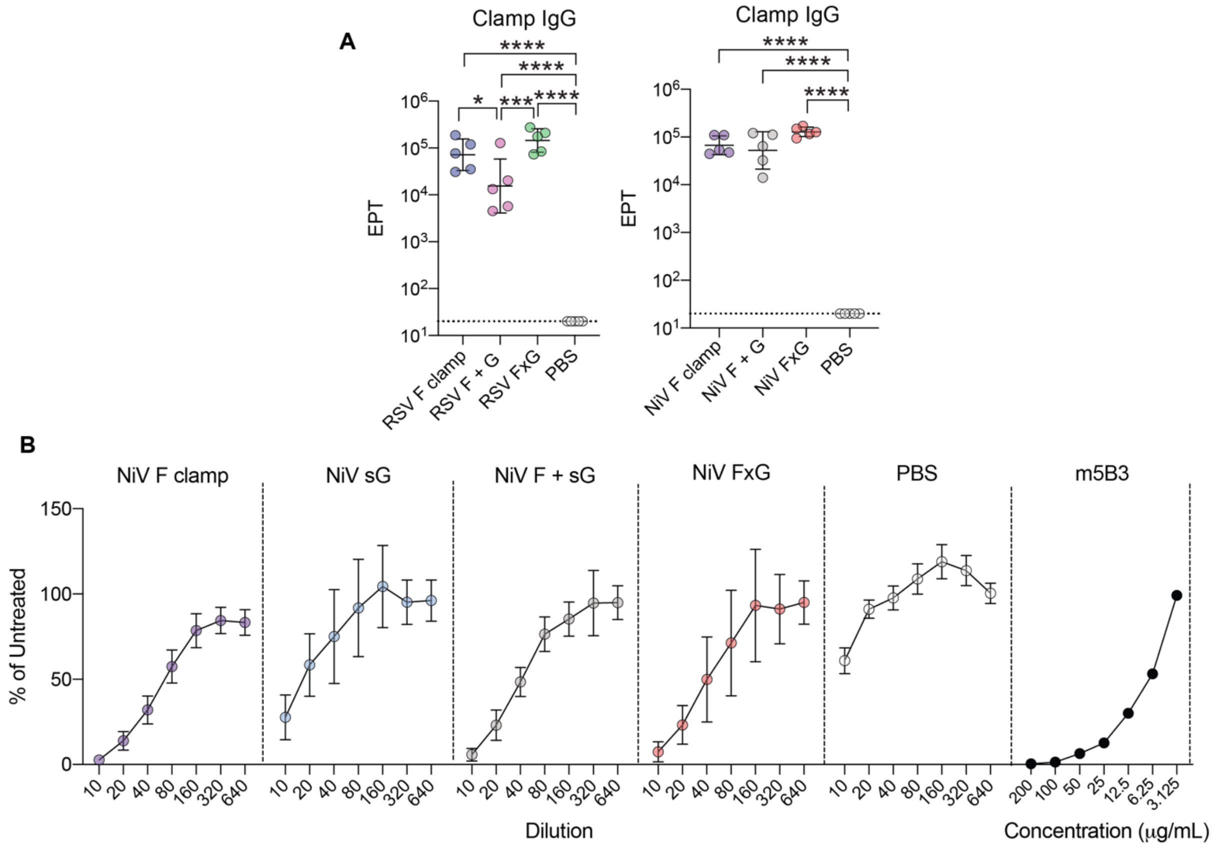

Figure S2. (**A**) Endpoint titer (EPT) of each RSV (left) and NiV (right) groups against the molecular clamp domain, assayed against a non-specific clamp-stabilised protein. Dotted line shows limit of detection and data is expressed as geometric mean with geometric standard deviation (SD). p-values calculated using a one-way Tukey's multiple comparison ANOVA on log transformed values, where * = $p < 0.05$, ** = $p < 0.005$, *** = $p < 0.0005$ and **** = $p < 0.0001$. (**B**) mFITs of NiV groups showing serial dilutions of sera from vaccinated groups as well as positive mAb controls. Each mouse sample was assayed in triplicate. Data is expressed as a percentage of the average luciferase readings seen in untreated controls (no serum) with 50% inhibition dotted line indicated (IC_{50}). Values shown are group mean (n = 5) with SD.

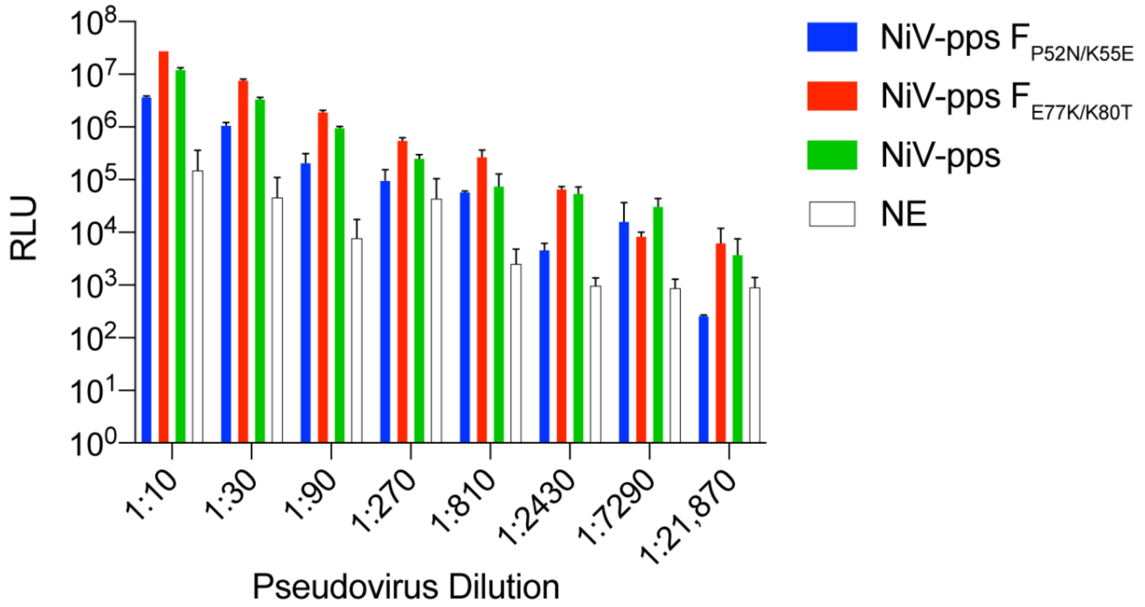

Figure S3. Titration of NiV pseudovirus (NiV-pps) wild-type or mutant F variants on BHK-21 cells. Titer is measured as relative light units (RLU) emitted by firefly luciferase reporter. Non-enveloped (NE) virus is included as background control.

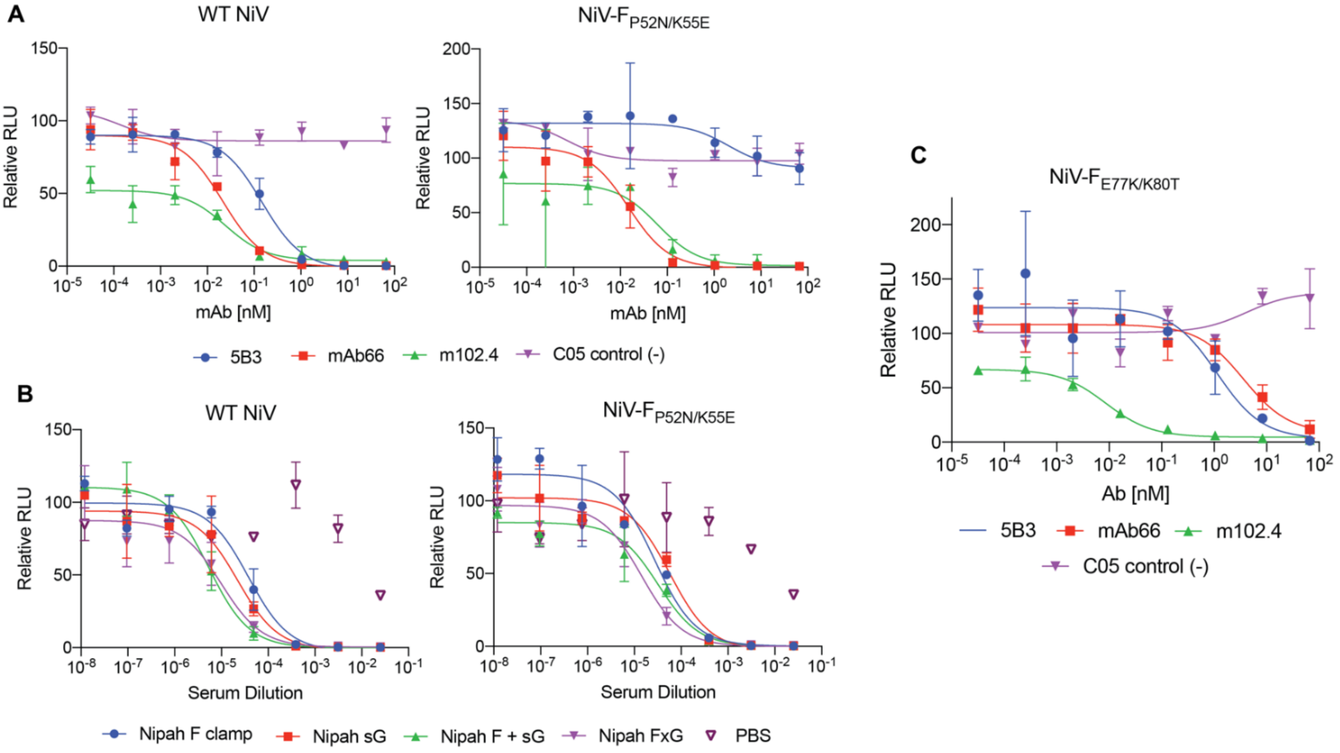

Figure S4. NiV pseudovirus neutralisation assays of mAbs (**A**) and sera (**B**) against wild-type NiV (WT) or NiV-$F_{P52N/K55E}$ mutant knockout. (**C**) NiV pseudovirus neutralisation assay of mAbs against NiV- $F_{E77K/K80T}$ mutant knockout. Data shown is duplicate values of pooled sera or duplicate values of mAbs expressed as percent inhibition relative to virus only readings (relative RLU). C05 is a non-specific antibody control.

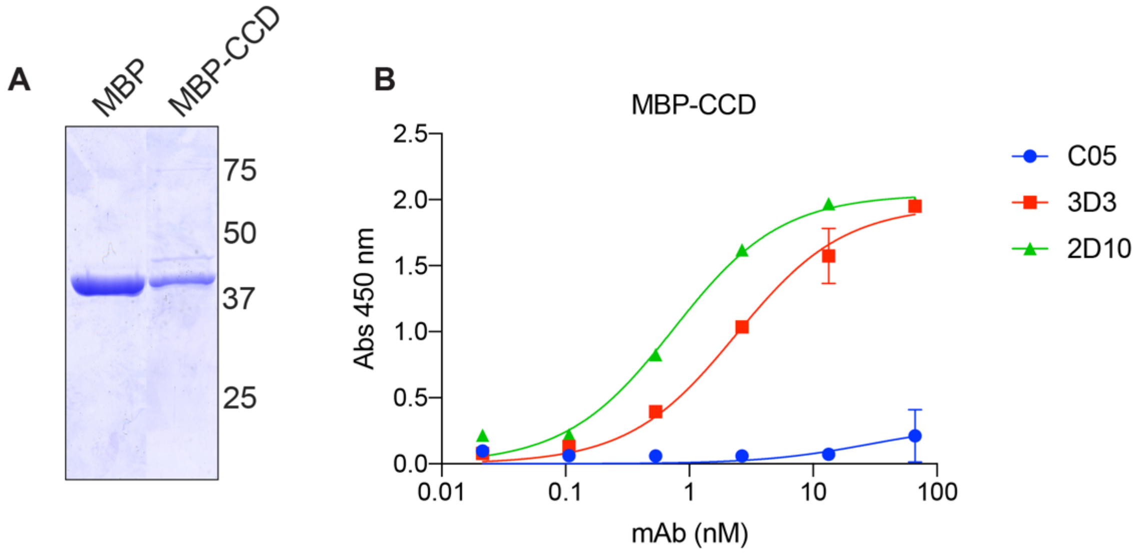

Figure S5. (**A**) SDS-PAGE of MBP and MBP-CCD purified protein run under reducing conditions. (**B**) Indirect ELISA of CCD-specific mAbs or non-specific control (C05) against MBP-CCD protein. Data shown is average of duplicate values with SD and background binding to MBP only subtracted.
